# Outcomes of Endovascular Stenting in Symptomatic Vertebral Artery Ostial Stenosis

**DOI:** 10.7759/cureus.21465

**Published:** 2022-01-21

**Authors:** Roy El Koussa, Jaime Graft, Sarah Linder, Vibhav K Bansal

**Affiliations:** 1 Internal Medicine, Javon Bea Hospital, Rockford, USA; 2 Interventional Neurology, Javon Bea Hospital, Rockford, USA

**Keywords:** drug eluting stent, endovascular stenting, angioplasty, extracranial vertebral artery, vertebral artery stenosis

## Abstract

Vertebral artery ostial stenosis is implicated in one-fifth of all posterior circulation cerebrovascular accidents. However, uniform treatment guidelines and data on the different treatment modalities are still lacking. Endovascular stenting is an emerging therapy for symptomatic vertebral artery ostial stenosis when medical management fails. This manuscript will examine the safety and efficacy of endovascular revascularization with drug-eluting stents in a series of ten consecutive patients that had failed medical management. We also report the rate of complications and in-stent restenosis, as well as the rate of recurrent cerebrovascular accidents.

## Introduction

Vertebral artery ostial stenosis is common amongst patients with cardiovascular risk factors [1]. Vertebral artery stenosis has long been thought to be inconsequential as most people have two vertebral arteries and patency of just one is enough for adequate circulation. However, it is now known that patients with unilateral vertebral artery stenosis have a stroke rate of 14% per annum [2]. Moreover, 20% of posterior circulation transient ischemic attacks and strokes are attributable to vertebral artery stenosis [3].

Although poorly studied, optimal medical management of symptomatic vertebral artery stenosis is the current treatment of choice [4]. However, when this fails, surgical or endovascular revascularization becomes necessary. Traditional treatment modalities include vertebral artery bypass and vertebral artery reconstruction [5, 6]. More recently, endovascular revascularization with angioplasty, bare metal stenting, or drug-eluting stenting has become more common. While angioplasty carries a low risk of complications, restenosis frequently occurs [7]. Of note, bare-metal stents also carry a high risk of restenosis [8, 9].

Endovascular stenting with drug-eluting stents (DES) is the mainstay for coronary interventions as rates of restenosis and neo-intimal hyperplasia are significantly lower [10]. The diameter of vertebral arteries is comparable to that of coronary arteries [11, 12]. Consequently, the use of drug-eluting coronary stents for vertebral artery ostial stenosis has been proposed as a treatment modality. This paper describes 10 consecutive patients with symptomatic vertebral artery ostial stenosis treated with the drug-eluting Resolute Onyx stent (Medtronic, Minnesota, USA) with favorable morbidity and mortality and long-term stent patency.

## Materials and methods

Patient selection

**Figure 1:**
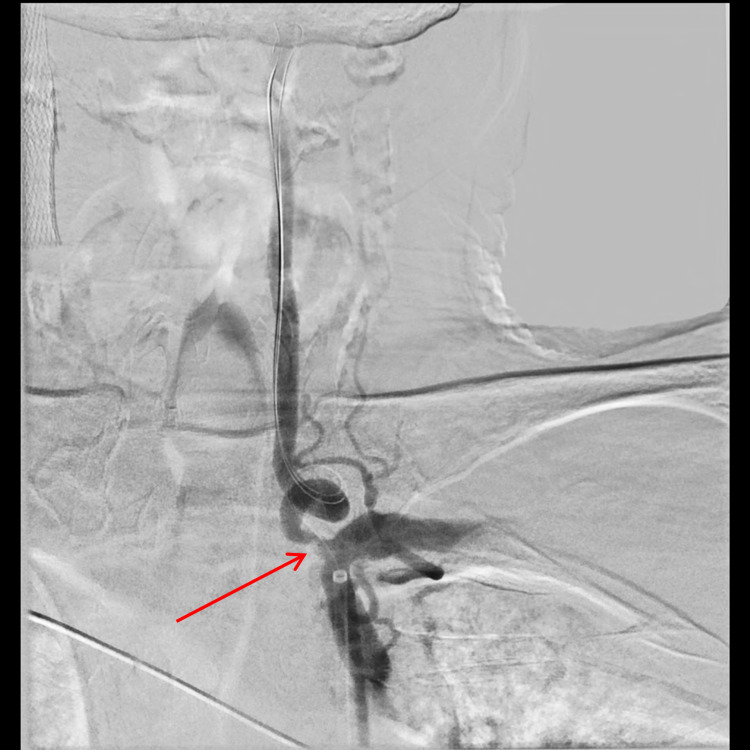
Angiogram showing greater than 50% stenosis of the vertebral artery (arrow)

This is a retrospective study of all patients who underwent endovascular stenting of the vertebral artery origin with Resolute Onyx stent between the years 2018 and 2021. A total of 10 patients were included in this study. Included patients had either confirmation of stroke isolated to the posterior circulation with magnetic resonance imaging (MRI) or transient ischemic attack (TIA) symptoms localizing to the posterior circulation as deemed by a stroke neurologist. All patients had a computed tomography angiography (CTA) or magnetic resonance angiography (MRA) demonstrating 50% or more stenosis of the dominant vertebral artery or bilateral vertebral artery stenosis greater than 50% (Figure 1). Patients were not excluded if they were not on medical therapy (i.e. antiplatelet therapy) for vertebral artery stenosis before undergoing endovascular revascularization.

Procedural technique

**Figure 2:**
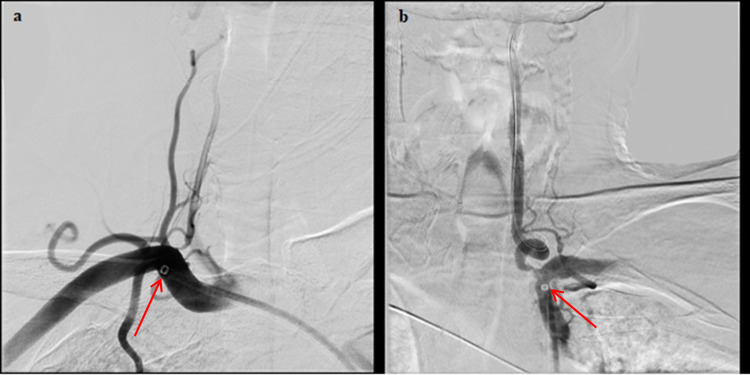
(a) Cook shuttle (arrows) parked distal to the vertebral artery origin during radial access approach and (b) proximal to the origin during femoral access approach

**Figure 3:**
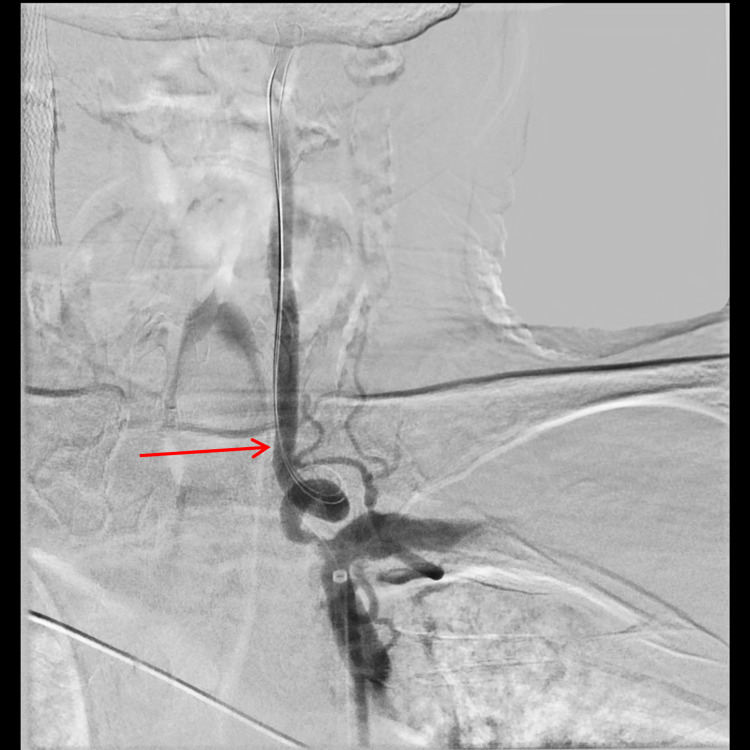
Stent sizing based on the vessel diameter (arrow) distal to the stenosis

Elective patients were started on Plavix for one week prior to intervention or given 600 mg load 24 hours prior to the procedure and subsequently started on daily dual antiplatelet therapy (Aspirin 81 mg and Plavix 75 mg) for one year. Acutely symptomatic patients were loaded with Plavix 600 mg and Aspirin 325 mg prior to the procedure. Heparin was given at the start of the procedure at a dose of 100 mg/kg to maintain an activated coagulation time > 250 seconds. The symptomatic vertebral artery was accessed via either the ipsilateral femoral or radial artery. A 6-french 80 cm Cook Shuttle (Medline Industries, Illinois, USA) was utilized with either approach. The shuttle was parked proximal to the origin of the vertebral artery within the subclavian artery for femoral arterial access and distal to the vertebral artery within the subclavian artery when the radial approach was undertaken (Figure 2a, 2b). Angiography was performed to confirm vertebral artery stenosis prior to stenting. Through the Cook Shuttle, a .014 synchro wire was gently advanced through the stenotic vertebral segment with a distal tip parked in the V2 segment of the vertebral artery. When necessary, pre-plasty with a Mini-Trek non-compliant balloon (Abbott, Illinois, USA) was performed. Stent sizing was based on the largest diameter of the normal segment of V1 distal to the stenosis (Figure 3). Under roadmap guidance, the Resolute Onyx stent was advanced to the stenotic segment; once adequately positioned, the stent was deployed at nominal pressure. Cervical and cerebral angiography was performed immediately post-deployment to ensure stent and basilar artery patency, respectively. All procedures were performed under conscious sedation. Aspirin and Plavix were continued for one year.

All patients were admitted to the neuro-critical care unit for 24 hours post-procedure for close observation.

Assessment of clinical and radiographical outcomes

All patients were seen in follow-up at six months and 12 months post-stenting. National Institute of Health stroke scale (NIHSS) and modified Rankin scale (mRS) were performed during each visit. Every patient underwent serial radiographic imaging, with either CTA or MRA, at six months and one year to evaluate stent patency. The imaging studies were independently reviewed by neuroradiology. Information regarding procedural morbidity, mortality, successful revascularization, recurrent stroke or TIA, and stent patency at follow-up was also collected.

## Results

Ten patients with symptomatic vertebral artery ostial stenosis were included in the study. Baseline characteristics of included patients are summarized in Table 1. 60% of patients had confirmation of stroke isolated to the posterior circulation by MRI and 40% suffered TIA localizing to the posterior circulation confirmed by a stroke neurologist. The median NIHSS before angioplasty was zero and remained unchanged during the six-month and 12-month follow-up visits. 

**Table 1:** Baseline Characteristics of Included Patients BMI: Body Mass Index - mRS: Modified Rankin Scale - NIHSS: National Institute of Health Stroke Scale - CAD: Coronary Artery Disease

Table 1: Baseline Characteristics of Included Patients	
Characteristic	Number (%)
Age – yrs.	59±13
Male sex	9 (90%)
BMI	29.5±2.6
mRS Baseline	
0-2	9 (90%)
3-4	1 (10%)
4-6	0 (0%)
NIHSS Baseline	
0	6 (60%)
1-4	2 (20%)
5-15	2 (20%)
21-42	0 (0%)
Comorbidities	
Diabetes	4 (40%)
Hypertension	8 (80%)
Dyslipidemia	9 (90%)
CAD	5 (50%)
Stenosis grade	
Moderate	1 (10%)
Severe	9 (90%)
Previous treatment	
Antiplatelet	8 (80%)
None	2 (20%)

The median mRS score at presentation and at six-month follow-up was one, going down to zero at 12-month follow-up (Table 2). All these patients underwent successful stenting of the stenotic vertebral artery. Three patients required pre-plasty prior to stent deployment. There was less than 10% stenosis of the offending artery at the conclusion of the procedure. There was no procedural or peri-procedural complication or stroke (defined as an increase in the NIHSS stroke score of at least one point). There was no other associated morbidity or mortality related to the procedure. All patients underwent follow-up MRA and/or CTA at six months and 12 months (Table 2). There was no in-stent restenosis. There was no recurrent stroke or TIA during the follow-up period.

**Table 2:** Patient's Characteristics at Follow-up MRA: Magnetic Resonance Angiography - CTA: Computed Tomography Angiography - mRS: Modified Rankin Scale - NIHSS: National Institute of Health Stroke Scale

Table 2: Patient’s Characteristics at Follow-up	
Characteristic	Number (%)
Follow-up Imaging	
MRA	1 (10%)
CTA	4 (40%)
MRA+CTA	5 (50%)
mRS Follow-up	
0-2	10 (100%)
3-4	0 (0%)
4-6	0 (0%)
NIHSS Follow-up	
0	7 (70%)
1-4	2 (20%)
5-15	1 (10%)
21-42	0 (0%)

## Discussion

Atherosclerotic vertebral artery ostial stenosis carries a significant risk of recurrent stroke [13]. Medical management remains the treatment of choice. In patients who have had recurrent stroke despite optimal medical management, the need for revascularization is important. Prior vertebral artery stenting trials yielded mixed results for a multitude of reasons. The carotid and vertebral artery transluminal angioplasty study (CAVATAS) trial, which enrolled 16 patients randomly assigned to receive either medical management or angioplasty, showed no difference between the two arms in terms of stroke incidence [14]. However, the sample size of the trial was very small and thus the results have limited applicability. It is important to note that there were no complications associated with the stenting procedure in the trial. The vertebral artery stenting trial (VAST) study, another vertebral artery stenting trial, demonstrated a higher risk of stroke in the stenting arm. However, these strokes were mainly observed in patients who underwent intracranial rather than extracranial/ostial stenting, again demonstrating the safety of stenting of ostial stenosis [15]. Moreover, the VAST study was underpowered and was stopped prematurely casting significant doubt about its findings. Vertebral artery ischemia stenting trial (VIST) trial, the most recent vertebral artery angioplasty trial, demonstrated a decreased risk of stroke in the angioplasty arm relative to the medical management arm however, the difference was not statistically significant. It’s important to note that most patients in the VIST trial had extracranial/ostial stenosis hence conclusions about the safety and efficacy of angioplasty of intracranial stenosis are not possible. Moreover, there were no procedural-related strokes in the VIST trial.

These studies underscore the safety of vertebral artery ostial stenting peri- and post-procedurally. As with endovascular coronary artery revascularization, treatment with angioplasty alone or with the use of bare-metal stents carries a higher risk of in-stent restenosis. With the introduction of DES, stent patency has significantly improved as DES has anti-proliferative effects, reducing neo-intimal hyperplasia.

The previous case series confirmed the safety and low rate of in-stent restenosis using several different DES for vertebral artery ostial stenosis [16-18]. The current study is unique in that it provides information on a consecutive series of patients treated exclusively with Resolute Onyx stent. All patients enrolled in this study had symptomatic stenosis. The procedural success rate of 100% without any complications (minor or major) underscore the safety of the procedure. The persistent patency of the stent during the follow-up period in all the patients is a testament to the durability of the procedure. Finally, the fact that no patient had a stroke or TIA during the follow-up period suggests the technique’s efficacy in treating symptomatic vertebral artery ostial stenosis.

This study has several limitations. Namely, the study was a single-arm study that was performed at a single center. There was no control group. Moreover, the sample size was small. Finally, patients were consistently followed for one year only, thus the possibility of delayed in-stent stenosis or recurrent stroke was not evaluated.

## Conclusions

Vertebral artery stenosis accounts for a significant proportion of all ischemic strokes. However, the management and treatment of symptomatic vertebral artery stenosis are poorly studied. Current treatment guidelines are based on a low level of evidence. In our study, all patients underwent endovascular stenting with an Resolute Onyx stent and were followed for one year after the intervention. None of our patients experienced adverse outcomes. All stents remained patent after one year, with no patient having a recurrent stroke or TIA. Our current study builds on previously published data and adds to the body of evidence that attests to the safety and efficacy of stenting in cases of symptomatic vertebral artery ostial stenosis. Large, multicenter studies are needed to corroborate and prove the external validity of our findings.
